# Biochemical and functional characterization of heat-inactivated coelomic fluid from earthworms as a potential alternative for fetal bovine serum in animal cell culture

**DOI:** 10.1038/s41598-024-56169-0

**Published:** 2024-03-07

**Authors:** Melinda Grace Rossan Mathews, Ravichandran Subramaniam, Saravanakumar Venkatachalam, Johnson Retnaraj Samuel Selvan Christyraj, Beryl Vedha Yesudhason, Kalishwaralal Kalimuthu, Manikandan Mohan, Jackson Durairaj Selvan Christyraj

**Affiliations:** 1 Regeneration and Stem Cell Biology Lab, Centre for Molecular and Nanomedical Sciences, International Research Centre, Sathyabama Institute of Science and Technology, Chennai, Tamil Nadu India; 2 Division of Cancer Research, Rajiv Gandhi Centre for Biotechnology, Thiruvananthapuram, Kerala India; 3 Wallace H. Coulter Department of Biomedical Engineering, Emory University and Georgia Tech, Atlanta, USA

**Keywords:** Earthworm, Coelomic fluid, *Perionyx excavatus*, Fetal bovine serum, Serum free media, Biochemistry, Biological techniques, Biotechnology

## Abstract

Fetal bovine serum (FBS) plays a pivotal role in animal cell culture. Due to ethical and scientific issues, searching for an alternative, comprising the three R’s (Refinement, Reduction and Replacement) gained global attention. In this context, we have identified the heat inactivated coelomic fluid (HI-CF) of the earthworm, *Perionyx excavatus* as a potential alternative for FBS. Briefly, we formulated HI-CF (f-HICF) containing serum free medium which can aid the growth, attachment, and proliferation of adherent cells, similar to FBS. In this study, we investigated the biochemical characterization, sterility, stability, formulation, and functional analysis of HI-CF as a supplement in culturing animal cells. Notably, vitamins, micronutrients, proteins, lipids, and trace elements are identified and compared with FBS for effective normalization of the serum free media. HI-CF is tested to be devoid of endotoxin and mycoplasma contamination thus can qualify the cell culture grade. The f-HICF serum free media was prepared, optimised, and tested with A549, HeLa, 3T3, Vero and C2C12 cell lines. Our results conclude that f-HICF is a potential alternative to FBS, in accordance with ethical concern; compliance with 3R's; lack of unintended antibody interactions; presence of macro and micronutrients; simple extraction; cost-effectiveness and availability.

## Introduction

Cell and tissue culture have gained an exponentially important role in basic and applied biomedical research. They were widely employed in the fields of cell biology, biotechnology, physiology, diagnostic studies, pharmacology, and toxicology. Animal serum supplementation to the basal culture medium is essential for the cell attachment, metabolism, growth, proliferation, and differentiation of the cells in vitro. The supplementation of Fetal Bovine Serum (FBS) in animal cell culture is inevitable for the growth and proliferation of the cells^1–3^. Issues revolving around the usage of FBS include global supply versus demand, higher cost, and scientific disadvantages such as quantitative and qualitative variations in their composition, contamination risks of endotoxin and microbial contaminants, undesigned interactions of antibodies, bio-safety issues such as exposure to prions and viral contaminants, batch to batch variability, reproducibility, geographical and seasonal variations^2–4^. FBS collection is unethical and painful for bovine fetuses. This involves removing the fetus from the dead mother, puncturing the fetal heart, and withdrawing blood, ultimately killing them. There is very little evidence that calves are being protected by guidelines^3^. In light of the above-mentioned issues, the search for the replacement of FBS in terms of the three R’s (Refinement, Reduction and Replacement) has gained global attention in the field of cell and tissue culture research^5^.

Due to ethical, scientific, commercial, and geographical concerns about FBS, serum free media (SFM) formulations were designed^6^. Insufficient knowledge of the mechanisms underlying serum's exceptional capacity to promote cell growth is one of the fundamental problems with replacing serum^7^. The coelomic fluid (CF) of the earthworm *Perionyx excavatus* was proven to be a potential alternative for FBS in animal culture. However, it lacks cell attachment due to the presence of fibrinolytic enzymes^8,9^. In this study, we have formulated a heat inactivated coelomic fluid (HI-CF) containing serum free media (f-HICF), which aids in the growth, attachment, and proliferation of the adherent cells. Coelomic fluid is obtained from *P. excavatus* and definite concentrations of certain attachment and growth factors were added to the DMEM medium. This cocktail mixture is given to the adherent cell lines and it is observed to aid in the attachment, proliferation, growth, and viability of the cells. The use of HI-CF in our formulated serum free media (SFM) represents a better alternative to use of FBS, in terms of cost-effectiveness, compliance with the 3Rs, lack of unintended antibody interactions, presence of macro and micronutrients, simple extraction, and availability. In this paper, the biochemical characterisation, sterility, and stability of the HI-CF, which is the primary component of the f-HICF and its growth promoting efficiency in various animal cell lines were studied.

## Results

### Elemental composition and biochemical characteristics of HI-CF

Coelomic fluid was collected from the earthworm, *P. excavatus* followed by heat inactivation and stored in a − 20 °C deep freezer. Parameters such as pH and colour were monitored regularly. The colour of the coelomic fluid was observed to be greenish yellow, and the pH was 7 (neutral pH), which is within the tolerance of cell lines and an acceptable range for cell culture media. Both the parameters remained unchanged. Biochemical analysis of HI-CF was performed and the amounts of potassium, chloride, sodium, iron, cholesterol, urea, creatinine, glucose and total protein were determined and compared with FBS as tabulated in Table 1.

**Table 1:** Biochemical composition of heat inactivated coelomic fluid compared with fetal bovine serum.

Component	Estimated amount in HI-CF	Estimated amount in FBS (Lindl^10^; Yang and Xiong^26^)
Potassium	0.0077 mmol/ml	11,000 mmol/ml
Chloride	0.2623 mmol/ml	103,000 mmol/ml
Sodium	0.1131 mmol/ml	137 mmol/ml
Glucose	772.5 mg/ml	1.25 mg/ml
Cholesterol	110 µg/ml	310 µg/ml
Urea	37 µg/ml	160 µg/ml
Creatinine	0.3 µg/ml	31 µg/ml
Total protein	0.00163 mg/ml	38 mg/ml
Iron	1.02 ppm	–

**Figure 1:**
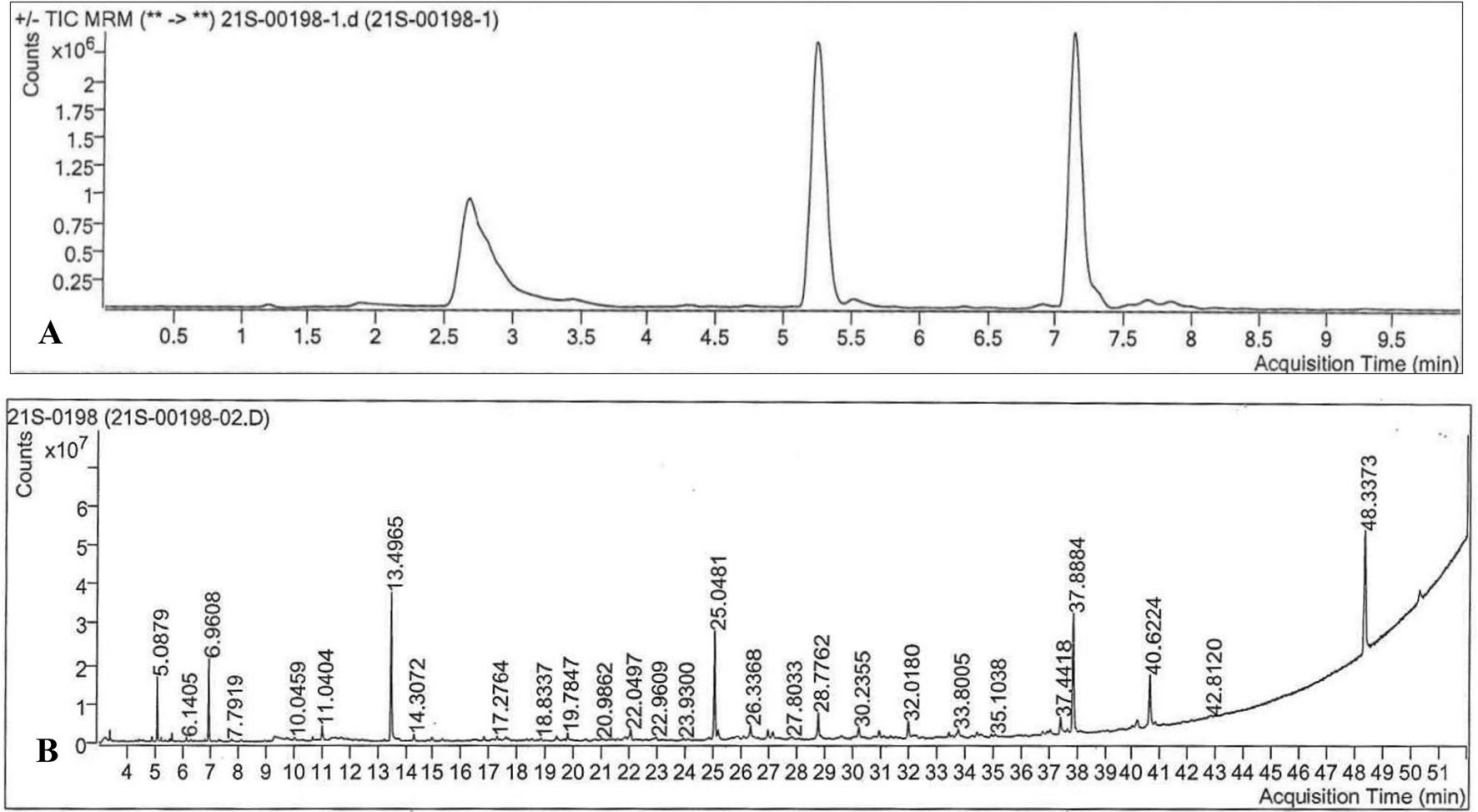
Vitamins, fatty acids, and trace elements present in heat inactivated coelomic fluid analysed using gas chromatography and mass spectrometry. (**A**) Vitamin chromatogram; (**B**) Fatty acids and trace elements chromatogram.

Vitamins such as nicotinic acid, niacinamide, pantothenic acid, cyanocobalamin, biotin and riboflavin were identified in the HI-CF. The estimated amount and retention time (RT) of the identified vitamins in the HI-CF were tabulated in Table 2 and the chromatogram is shown in Fig. 1A. Using GC–MS, lipid and trace elements profiling was analysed, and totally 70 compounds were identified, of which known compounds along with their molecular formula and structure were tabulated in the supplementary table S1. Corresponding chromatogram is shown in the Fig. 1B.

**Table 2:** Estimated amount and retention time of the identified vitamins in the heat inactivated coelomic fluid.

Vitamin	Estimated amount (in ng/ml)	Retention time (in min)
Nicotinic acid (vitamin B3)	93.87	1.953
Niacinamide (vitamin B3)	79.8140	2.773
Pantothenic acid (vitamin B5)	–	5.247
Cyanocobalamin (vitamin B12)	0.8295	6.232
Biotin (vitamin B7)	6.895	6.916
Riboflavin (vitamin B2)	–	7.138

### Stability of HI-CF composition during storage

**Figure 2:**
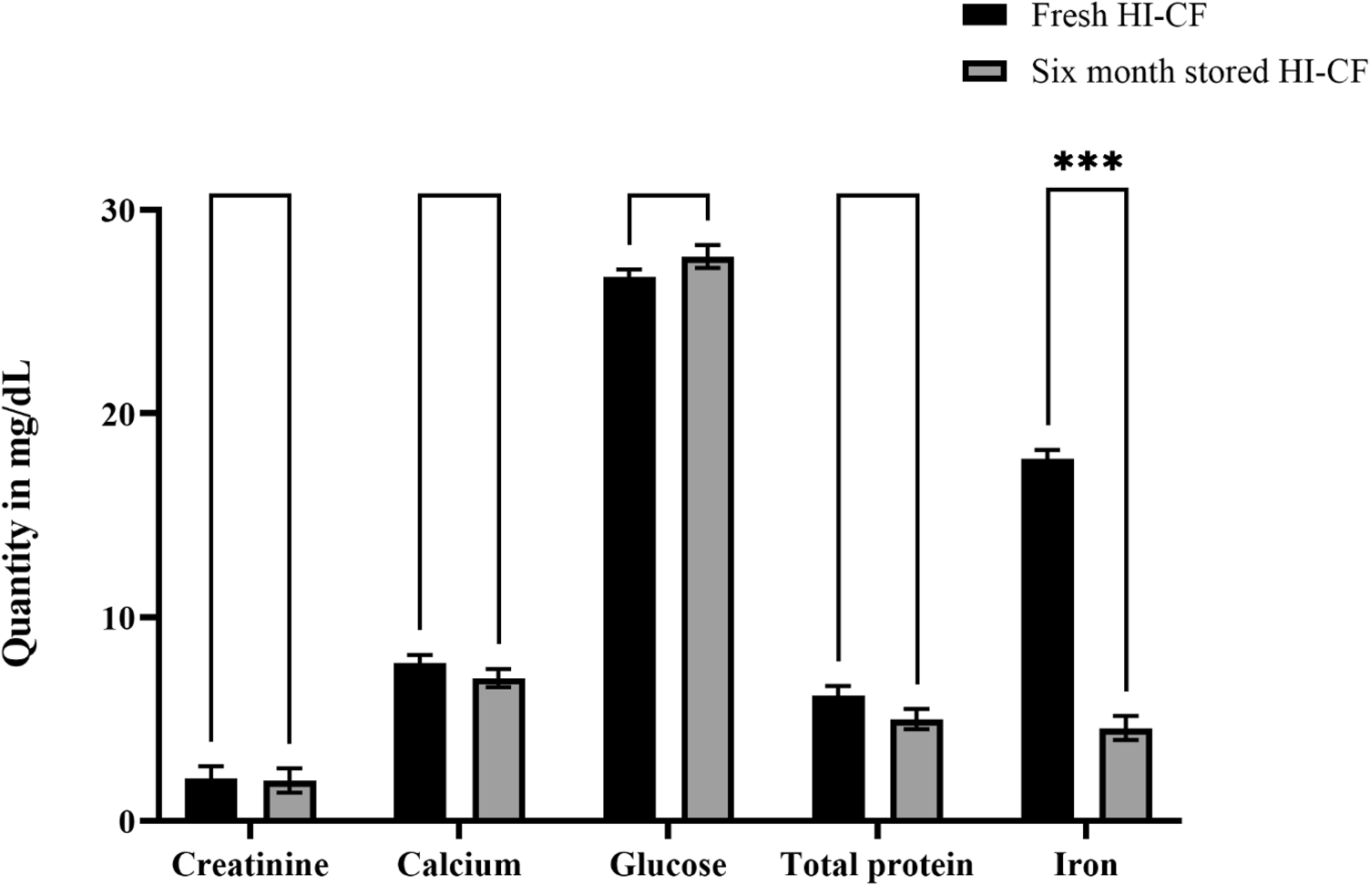
Stability of the elemental quantity of fresh and 6-month-old HI-CF stored in − 20 °C. Data indicate the average value of triplicates (mean ± SD). ***Significant difference p < 0.001.

The effects of storage on the stability of HI-CF were studied by quantifying the elemental composition of fresh and 6 months stored HI-CF at − 20 °C. Based on the results, there was a slight change in their elemental quantity with creatinine, calcium, glucose and total protein which was plotted in the graph (Fig. 2). Iron was significantly decreased upon storage.

### Endotoxin and mycoplasma test

**Figure 3:**
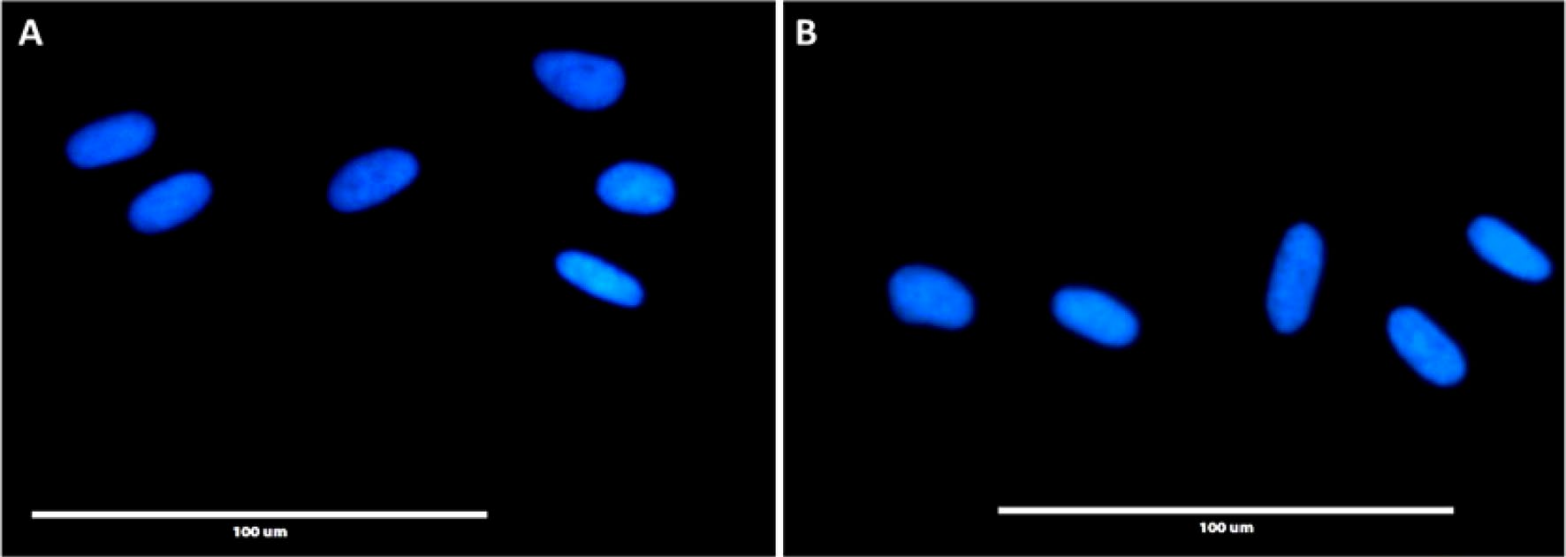
Mycoplasma detection assay using DAPI staining method for FBS (**A**) and HI-CF (**B**) supplemented cells.

Endotoxin testing was performed using an FDA approved gel clot approach using a variant of the *Limulus* Ambeocyte Lysate (LAL) method. At 0.125 EU/ml sensitivity, crude coelomic fluid (C-CF) showed clot formation whereas in the diluted C-CF in 1× PBS, no clot was formed. HI-CF showed a negative result (no clot formation). Mycoplasma contamination was detected through DAPI staining. Both the FBS and HI-CF shown no signs of mycoplasma ie., no appearance of particulate or filamentous staining over the cytoplasm of the cells (Fig. 3).

### Optimisation of HI-CF for cell culture

Different types of cell growth factors and attachment factors were used with HI-CF to design a serum free medium. The formulated HI-CF (f-HICF) serum free media was prepared with definite concentrations of HI-CF, selenium fetuin, insulin, and transferrin in DMEM media after testing with different factors.

### Comparison of cells cultured in f-HICF and a commercial serum free media

**Figure 4:**
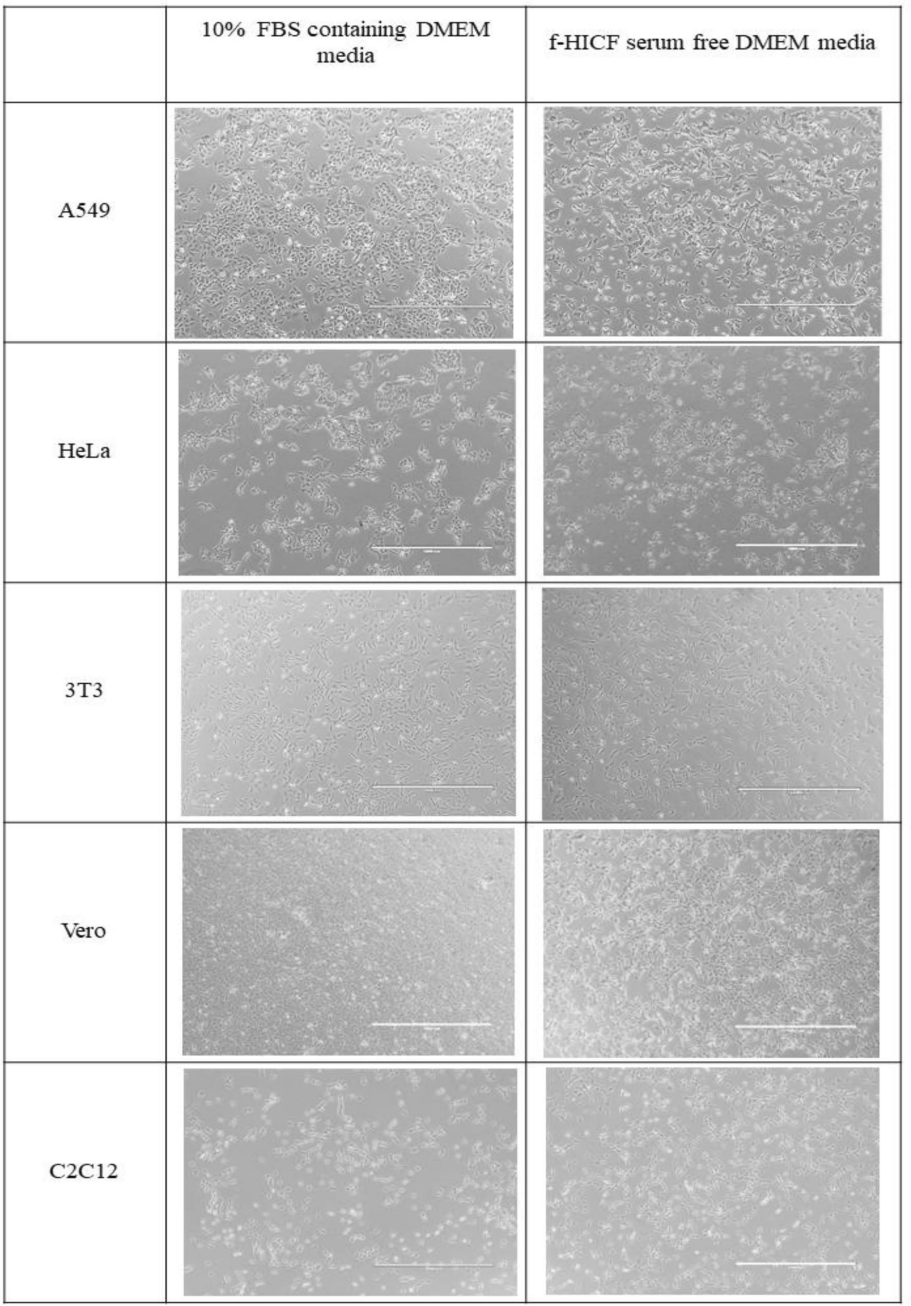
Cell lines cultured in 10% DMEM media and f-HICF serum free media.

**Figure 5:**
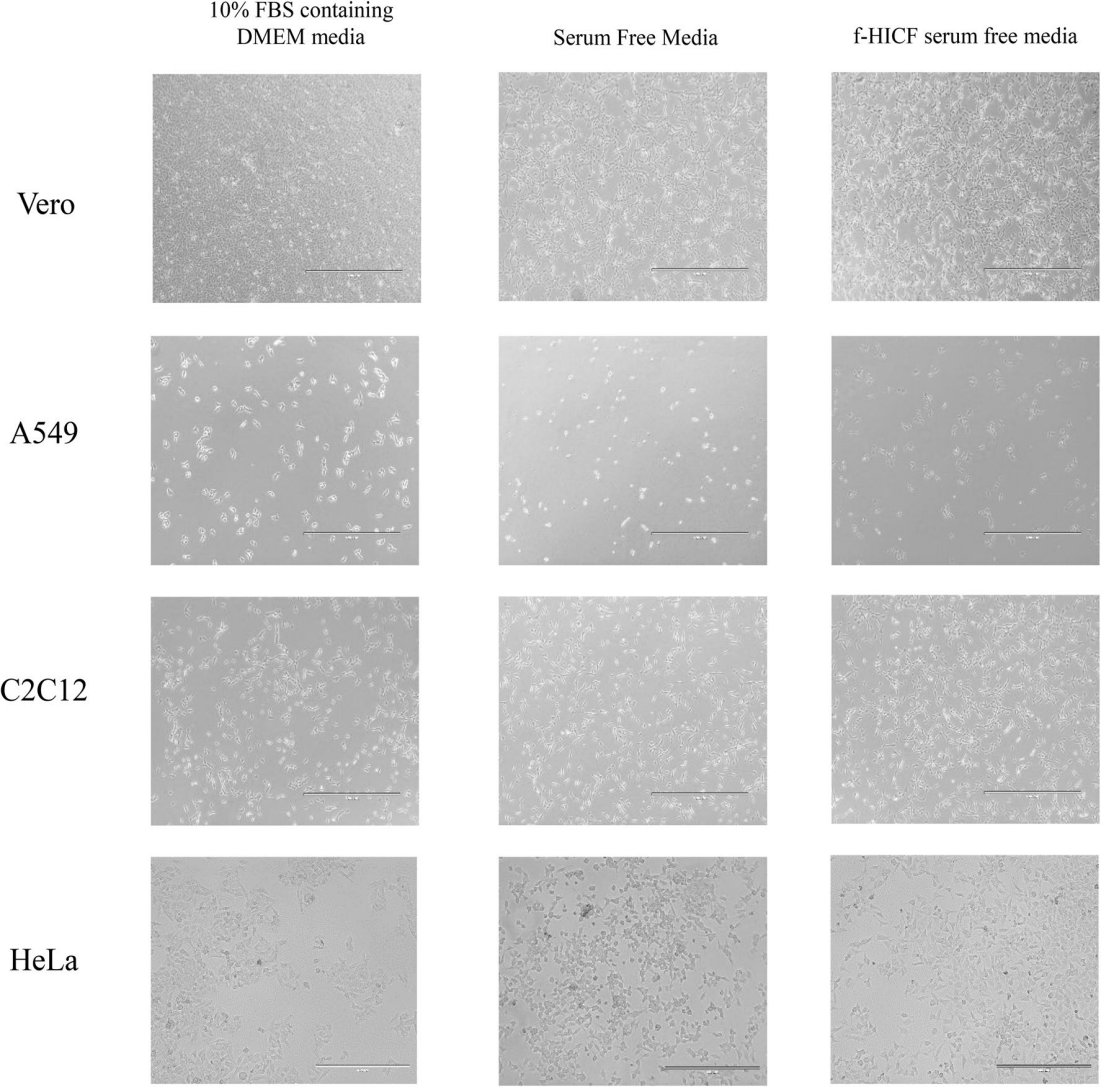
Cell lines cultured in 10% DMEM media, commercially available serum free media (SFM) and f-HICF serum free media.

Interestingly, cells (A549, HeLa, 3T3, Vero and C2C12) were supplemented with f-HICF media, they have shown significant cell growth, proliferation and attachment in the flask compared to FBS. The integrity of the cells with each other and to the surface, in control media and in formulated HI-CF serum free media was found to be the same. All cells exhibited adherence to the culture vessel and no early deattachment of cells was noticed. Upon morphological observation, A549, HeLa and Vero maintained their epithelial like morphology, while 3T3 and C2C12 maintained their fibroblast and myoblast nature respectively throughout their exposure to f-HICF supplementation. This assess the cell attachment and growth promoting nature of f-HICF in animal cell lines. Their attachment, growth and morphology were observed in comparison with cells maintained in 10% FBS containing DMEM media as shown in Fig. 4. Notably, compared with SFM, f-HICF supplemented cell lines exhibited good attachment, proliferation and growth (Fig. 5). The growth rates of cells cultivated in f-HICF were found to be similar to those cultured in FBS. In contrast, the cells cultured in SFM exhibited a consistently reduced rates of proliferation as compared to those cultured in FBS containing DMEM media and f-HICF. The f-HICF had an efficacy of roughly 70%, whereas the SFM revealed only 50% effectiveness compared to FBS in the cell culture experiments conducted. However, when considering cell morphology, it was shown that cells cultivated in serum-free medium (SFM) exhibited significant increase in cytosolic vacuoles and granularity compared to the other two test media.

### Plating efficiency of cells cultured in different media

**Figure 6:**
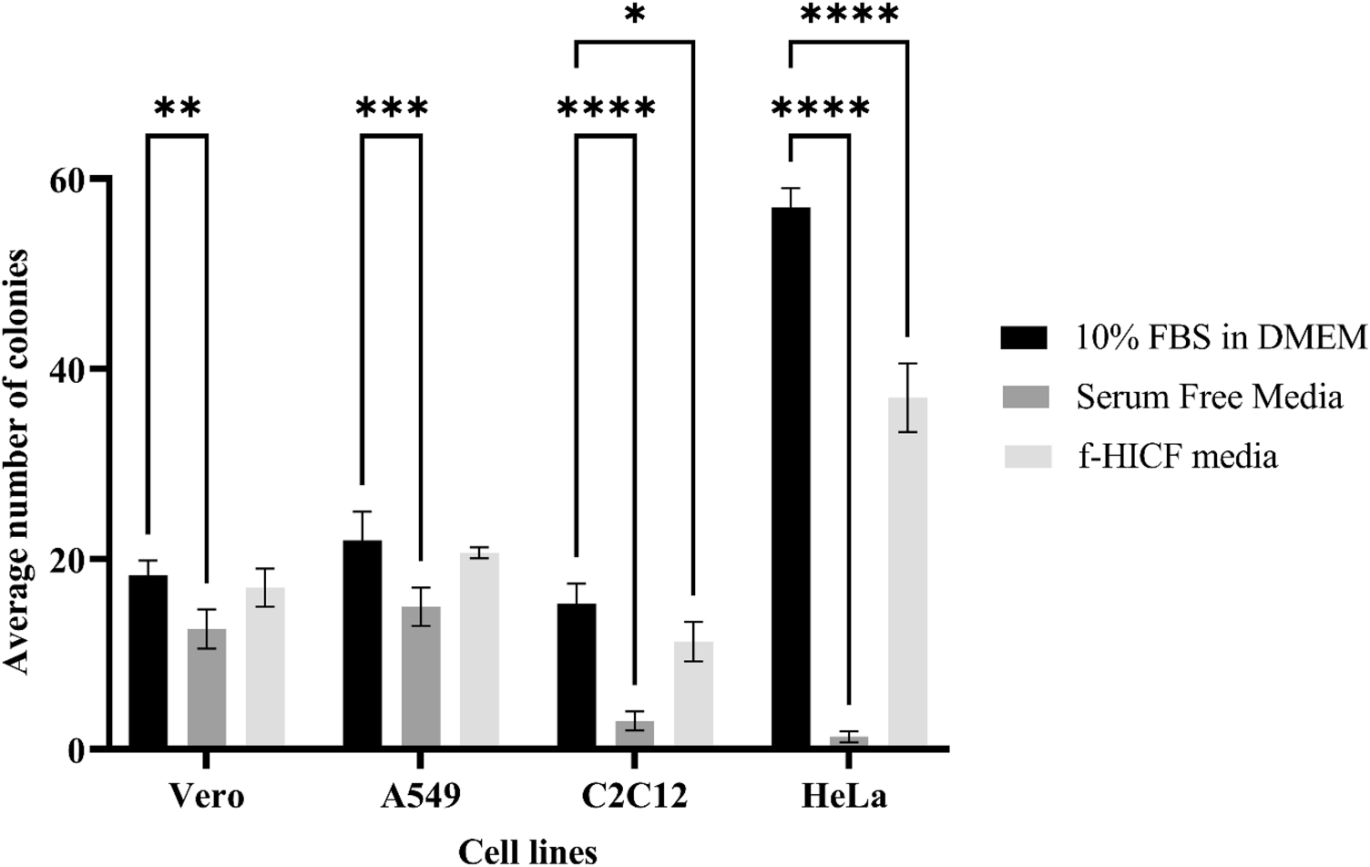
Plating efficiency of cell lines (Vero, A549, C2C12 and HeLa) cultured in 10% fetal bovine serum (FBS) containing DMEM media, commercially available serum free media and formulated heat inactivated coelomic fluid (f-HICF) serum free media. Data indicate the average value of triplicates (mean ± SD). *Significant difference from control (p < 0.05), **Significant difference from control (p < 0.01), ***Significant difference from control (p < 0.001) and ****Significant difference from control (p < 0.0001).

The plating efficiency of the cell lines in the test medium compared with the 10% FBS supplemented DMEM medium and commercially available SFM was evaluated to test their ability to promote cell growth at very low cell densities. Based on the results (Fig. 6), 10% FBS has superior efficiency in supporting cell growth and colony formation at low cell density followed by f-HICF and SFM media in all four tested cell lines.

### Functional analysis of batch variability in f-HICF

Based on the result (Supplementary data S2), different batches of f-HICF serum free media have shown very minimal variability in the context of functional role. Meanwhile, their biochemical variability will be extensively studied in future studies.

## Discussion

In this study, the physical, biochemical, and functional properties of HI-CF as a serum alternative were explored. According to Lindl^10^, FBS has an average pH of 7.4. One among the major issues with serum free media is the low pH and absence of pH buffering capacity which affect the growth kinetics of the cell^11,12^, which is mitigated in formulated HICF (f-HICF) media. Based on our research findings, employing the heat shock method allows for the extraction of a maximum of 140 ml of HI-CF from 1 kg of adult worms. Additionally, it has been observed that worms can replenish their coelomic fluid within a span of 15 days after the extraction process. As a result, this procedure is scalable and recurrent. Thus, practically it is possible to generate large amounts of coelomic fluid from earthworms.

Vitamin profiling of HI-CF shows that six vitamins were present, which provides a base support for acting as an alternative supplement for FBS in animal cell culture media. Briefly, Riboflavin aids in many metabolic and enzymatic processes as a cofactor (FMN and FMD) for redox processes^13^. Cyanocobalamin acts as a coenzyme in the metabolism of propionate and amino acids. Biotin is a coenzyme that functions in the citric acid cycle, fatty acid synthesis and catabolism of branched chain amino acids^13^. Pantothenic acid is a coenzyme precursor acts as an acyl group carriers and carboxyl group activators^13^. Nicotinic acid is converted into NAD and NADP coenzymes in vivo which play an active role in many enzymatic and energy producing cycles^13,14^.

There is a lack of knowledge about the components of serum and its attributes for cell attachment and proliferation^15^. Thus it is very important to understand the composition of FBS alternatives to mitigate these disadvantages. Most of the biological alternatives have unknown composition and it alters based on the genetic variation of the source, leading to batch variability. Eventhough the quantity of the HI-CF components highly differs from the golden standard of animal cell culture FBS, yet it provides a sufficient degree of nutrients for the cells to proliferate. Batch and price variability is common in FBS since it is reared from different groups of cattle which may have different diet, genetic diversity and from different location^16,17^. Even a small variation in the FBS compositon is greatly reported to affect the phenotypical properties of cell culture^18,19^. Further, the stability in the elemental composition of fresh and 6 months stored HI-CF shows negiligible variation, when stored at − 20 °C, proves that it could be a better alternative compared with FBS and SFM.

FBS is known for its unethical way of collection which causes pain and death of bovine fetuses. A syringe is used to draw blood from the beating heart of the calf fetus. This implies that fetal calves may potentially experience pain and discomfort during the process of collecting FBS. This includes the removal of the fetus from the dead mother's body, the puncturing of fetal calf heart, and the subsequent withdrawal of blood, ultimately resulting in their death. Despite the existence of suggestions aimed at preventing the suffering of calves, there is only limited evidence to suggest that these recommendations are being implemented^3,20^. However, HI-CF is collected non-invasively from the earthworms and hence there is a 100% survivability of the organisms after the extraction of coelomic fluid. No worms were reported dead or fatally wounded during CF extraction. Due to minimal shock, worms have shown very slow movements and they slowly regain their normal activity within 2 h. HI-CF adheres to the principles of the 3R’s: replacement—it can replace the serum derived from vertebrates (FBS) with the coelomic fluid obtained from invertebrates (HI-CF), which has a lesser sensitivity for perceiving pain; reduction—it can reduce the need for fetal slaughter for animal cell culture; and refinement—it refines the process of collecting CF non-invasively as a serum substitute in animal cell culture.

FBS undergoes various tedious processes to ensure cell culture grade safety and to pass the quality control. Yet, there is a huge possibility of contamination while processing, aliquoting, storing and shipping. Moreover the safety of laboratory personnel who handles FBS is a question, they may get exposed to the prions, viral contaminants and endotoxins^3^. In case of HI-CF, it is very simple to extract from earthworms, animal cruelty free, easy to sterilize through filtering and does not require any tough quality control testing, sophisticated instruments or techniques or trained staffs. The price and availability of FBS fluctuates in accordance with livestock numbers, regulations on importing, weather conditions, meat and livestock feed costs. Also, there is no monetary benefits to the cattle ranchers^15^. In the case of HI-CF, the production cost is much cheaper than the FBS which would be beneficial for the farmers and small scale agroindustries. Human derived alternatives such as human serum and human platelet lysate were regarded as a possible solution but has disadvantages when it comes to cell proliferation and differentiation^1^. One notable benefit of utilising human serum-derived supplements is their non-xenogenic nature when used in human cell lines. However, as a result of their limited accessibility and exorbitant costs applications are primarily restricted to the cultivation of human cells for therapeutics^24^.

Endotoxin testing is one among the eligible criteria for the acceptance of an aseptic production of a biological product on a commercial scale. The LAL assay was preferred as it is a globally recognized method that provides faster and more sensitive results than other existing options. Some species of mycoplasmas can originate from FBS which causes contamination to the cell lines^21^. Our results have proven that HI-CF is void of endotoxin and mycoplasma, thus can qualify the cell culture grade easily without any extensive downstream processes. Positive result in the C-CF was due to the presence of higher concentration of protein in the sample, meanwhile those proteins break down during heat inactivation giving the absolute result for the endotoxin testing in the HI-CF.

Although the cell density and proliferation are lower in HI-CF supplemented cell lines when compared to FBS, yet the cells tend to grow and proliferate in HI-CF containing media. Another disadvantage observed in HI-CF supplementation is the deattachment of the cells within days^8^. To increase the efficiency of HI-CF as the serum alternative, growth factors were added and a formulated HI-CF (f-HICF) supplementation was standardized. Initially gelatin was tested with HI-CF by coating it in the flask. Eventhough the cells were attached, cells were appeared to be attached in clumps, which affected its natural colony morphology. Collagen type I derived from the rat tail was used at different concentrations along with HI-CF in 3T3 cell lines, but no significant attachment was observed. Collagen's inability to function as an attachment factor can be attributed to the absence of high molecular weight proteins in HI-CF, which mediate the function of collagen. Fetuin is the protein component derived from the serum is extensively studied for its function as attachment and it was supplemented in various concentrations to the cells along with HI-CF. Fetuin has provided effective cell attachment when compared to other attachment factors. Eventhough cell attachment was achieved using fetuin, to enhance cell growth and proliferation along with HI-CF and fetuin, other growth supplements were tested. A combination of selenium, insulin and transferrin was tested with HI-CF and fetuin in definite concentrations for culturing the cells. They have shown the best results and took further for the study. BSA was incorporated with this formulated medium for protein supplementation but the cells have not shown any effective differences.

Serum free medium is preferred for the culture of stem cells, cell therapy and cells used for vaccine and recombinant protein production^22,23^. An universal serum free media is nearly impossible based on the fact that different cell lines require different nutritional supplements^18^. So far, the formulated HI-CF containing serum free media has supported five different adherent cell lines—A549 (lung carcinoma epithelial cell line), HeLa (cervical cancer epithelial cell line), 3T3 (fibroblast cell line), Vero (Kidney cell line) and C2C12 (myoblast cell lines). These cell lines were maintained in the f-HICF serum free media. Gradual FBS weaning process is required for the adaptation of cells in most of the commercially available serum free media^18,24,25^ but f-HICF does not require these adaptations with the above mentioned cell lines. One of the major setbacks in the f-HICF serum free media is its ability to inactivate the trypsin while subculturing the cell lines. This could be bypassed by using the trypsin neutraliser or animal origin free trypsin like protease enzyme reagents which do not require any inactivation using serum. Our future prospect is to develop this f-HICF for different types of media, cell lines, and cell culture applications.

To conclude, based on this biochemical characterisation and experimentation with the cell lines, it is proven that HI-CF can be a potential alternative to reduce or replace the FBS usage in animal cell culture. In addition, HI-CF has many advantages over the FBS and other serum substitutes such as absence of antibodies, broad spectrum antibacterial activity against Gram positive and negative bacterium, less cellular cytotoxicity and capability to support versatile cell lines, very minimal batch to batch variability making it a potential universal serum alternative.

## Materials and methods

### Vermiculture and maintenance

*P. excavatus* was collected with permission from a SK farm at Tambaram, Chennai, Tamilnadu, India. Vermicultures were maintained at ‘Regeneration and Stem cell Biology laboratory, International Research Centre (IRC) at Sathyabama Institute of Science and Technology, Chennai, Tamil Nadu, India’. The earthworms were maintained at 22–26 °C in plastic tubs filled with substrates composed of cow dung, organic wastes, soil and leaf litters. The vermicompost will be removed and replaced with fresh soil and substrates at intervals of 10 days.

### Heat inactivated coelomic fluid preparation

Mature earthworms with the body mass of 0.5–0.8 g were taken for the collection of coelomic fluid (CF). Briefly, worms were washed thrice with distilled water and subjected to gut cleaning process. During this process, earthworms were incubated inside wet tissues containing box for 24 h. At the end of the incubation, coelomic fluid was extracted using the electric shock method. Electric shock was passed through electrodes using a power supply. Earthworms were excited at 10 V and as a response, coelomic fluid was extruded from their dorsal pores Coelomic fluid will be diluted using 1× PBS and subjected to heat inactivation at 90 °C for 5 min in dry bath and filtered with 0.2 µm syringe PVDF filter^8,9^.

### Elemental compositional analysis and biochemical characterisation

Elemental composition of HI-CF (Potassium, Chloride, Sodium) was determined using atomic absorption spectrometry (AAS). Amount of glucose in HI-CF was calculated iodimetrically in a weak alkaline medium. Iron, cholesterol, creatinine, and urea levels were determined using the biochemical analyser (Mindray BS-120). Total protein present in HI-CF was estimated using Lowry’s method. Bovine serum albumin (BSA) was taken as the standard and the absorbance was measured at 660 nm in UV–Vis spectrophotometer. Gas Chromatography Mass Spectrometry (GCMS) analysis was performed using Agilent 7000D GCMS for the identification of lipids and trace elements in HI-CF. Vitamins were profiled and quantified using GC–MS/MS triple quadrupole (QqQ) in multiple reaction monitoring (MRM) mode.

### Stability of HI-CF composition during storage 

HI-CF was tested for its stability in fresh and 6-month-old sample stored at − 20 °C by checking its compositional quantity. Compositional parameters such as creatinine, calcium, glucose, total protein, and iron quantities were checked through alkaline picrate method, arsenazo II method, GOD/POD method, Biuret method, and ferrozine/magnesium carbonate method respectively.

### Endotoxin and mycoplasma testing

Endotoxin testing was performed on the crude coelomic fluid and heat inactivated coelomic fluid using the traditional gel clot LAL assay kit from Lonza (F245-125SA). Mycoplasma contamination was detected using DAPI staining in animal cells. FBS (Control) and HI-CF (Test) supplemented cells were stained with 4′,6-diamidino-2-phenylindole (DAPI) and observed under the inverted fluorescent microscope (EVOS FL inverted digital fluorescence microscope).

### Optimisation of HI-CF serum free medium using different attachment and growth factors

Attachment factors and growth supplements such as gelatin, transferrin, collagen type I, insulin, bovine serum albumin (BSA), selenium and fetuin were used for the development of formulated HI-CF (f-HICF) serum free media for culturing 3T3 cells.

### Animal cell culture studies with f-HICF

The formulated HI-CF (f-HICF) serum free media was prepared with definite concentrations of HI-CF (1%), selenium (0.0633 µM), fetuin (0.123 mM), insulin (1.726 µM), and transferrin (15.177 µM) in DMEM media. Cell culture studies were performed on five different cell lines—A549 (CCL-185), Henrietta Lacks cells—HeLa (CCL-2), 3T3 (CCL-92), Vero (CCL-81) and C2C12 (CRL-1772). These cell lines are obtained from National Centre for Cell Science (NCCS), Pune, India. Prior to the experiment, cells were maintained in 10% FBS (Origin-Brazil; Gibco) supplemented Dulbecco’s Modified Eagle’s Medium (DMEM; Sigma-Aldrich) and no gradual serum starvation or adaptation was performed. Cell lines A549, HeLa, 3T3, Vero and C2C12 were directly transitioned to f-HICF supplemented DMEM media. Cells were seeded at a density of 0.05 × 10^6^ in 24 well plates and maintained in humidified atmosphere with 5% CO_2_ at 37 °C. The growth promotion capacity of the f-HICF supplemented media was assessed in comparison with 10% FBS containing media by analysing the attachment, growth, and cell morphological features such as shape, size, and appearance.

### Comparison of cells cultured in f-HICF and a commercial serum free media

HeLa cells, A549 cells, C2C12 cells and Vero cells were used for the comparison of f-HICF and a commercially available serum free medium (SFM). The cells were grown at the density of 0.05 × 10^6^ in 24 well plates at 37 °C in a 5% CO_2_ environment and observed under an inverted microscope (EVOS fl inverted digital fluorescence microscope; Advanced Microscopy Group). The attachment, growth, and morphological characteristics of the cells, such as shape, size, and appearance were studied.

### Plating efficiency of cells cultured in 10% FBS, f-HICF and SFM

HeLa cells, A549 cells, C2C12 cells and Vero cells were seeded in 24 well plate at a seeding density of 0.05 × 10^4^ containing control (10% FBS, SFM) and test medium (f-HICF). Then the cells were observed for their colony morphology at day 2. After 4 days of growth, 1 ml of respective fresh media was added to the well. On 8th day, cells were washed with 1× PBS and fixed with methanol. Fixed colonies which have greater than 64 cells were considered and the graph was plotted to study the growth trendline of the cells in different medium. Experiments were performed in triplicates.

### Functional analysis of batch variability in f-HICF

C2C12 cells were seeded in 24 well plate at a seeding density of 0.05 × 10^6^ containing control (10% FBS containing DMEM media) and test (f-HICF: batch 1, 2 and 3). All three batches of f-HICF contains biological replicates of HI-CF taken from different batches of *P. excavatus* and prepared individually. Cells were monitored for 48 h using an automated live cell imaging system (Celloger Mini Plus, Curiosis Inc., South Korea) at 10× Objective, bright field and the graph was plotted to study the growth trendline of the cells in different medium. Experiments were performed in triplicates.

### Statistical analysis

Results are presented as mean ± SD, and analysed using one-way ANOVA (Dunnett’s multiple comparison test) and multiple *t*-tests was calculated using GraphPad Prism 9.5.1. Differences were considered significant at p < 0.05. The bar diagram was plotted to depict the data graphically.

### Supplementary Information

Supplementary Table S1.

Dataset S2.

## Data Availability

The authors confirm that the data supporting the findings of the study are available in the article and its supplementary material. The complete formulation of the medium used in this study are included in this published article.
