# Supplementary material for: Biochemical and functional characterization of heat-inactivated coelomic fluid from earthworms as a potential alternative for fetal bovine serum in animal cell culture
Source: Sci Rep. 2024 Mar 7;14:5606. doi: 10.1038/s41598-024-56169-0 (PMC10920628; doi:10.1038/s41598-024-56169-0)
Supplement: Supplementary file 1 — Supplementary Table S1. [file 41598_2024_56169_MOESM1_ESM.docx]

**Supplementary table S1:** Identified compounds present in HI-CF and its retention time (RT), molecular weight, molecular formula and structure.

| **Sl.No** | **Compound Name** | **RT** | **Molecular Weight (g/mol)** | **Molecular Formula** | **Molecular Structure** |
| --- | --- | --- | --- | --- | --- |
| 1 | 2,2,3,4-tetramethylpentane | 3.37 | 128.25 | C_9_H_20_ | 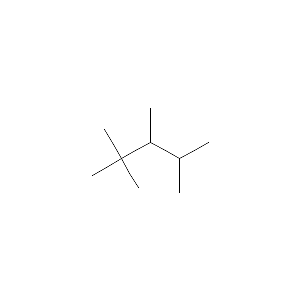 |
| 2 | Pentedrone | 4.43 | 191.27 | C_12_H_17_NO | 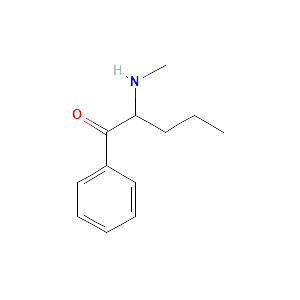 |
| 3 | Decane | 5.08 | 142.28 | C_10_H_22_ | 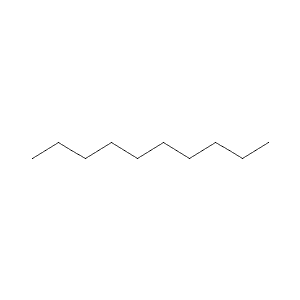 |
| 4 | 1,3,5-Cycloheptatriene, 3,7,7-trimethyl- | 5.53 | 134.22 | C_10_H_14_ | 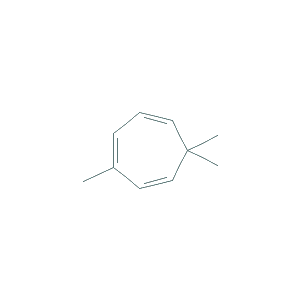 |
| 5 | 1,5-dimethyl-6-methylenespiro[2.4]heptane | 5.61 | 136.23 | C_10_H_16_ | 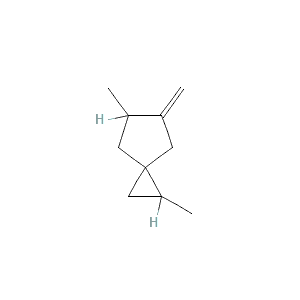 |
| 6 | 2,2,5,8,8-pentamethyl-5-[(trimethylsilyl)oxy]-3,7dioxa-2,8-disilanonane | 6.14 | 322.66 | C_13_H_34_O_3_Si_3_ | 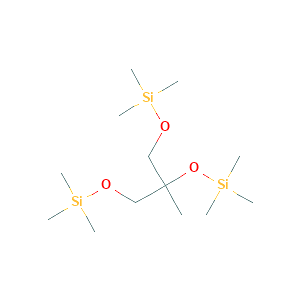 |
| 7 | (+)-4-Carene | 6.3 | 136.23 | C_10_H_16_ | 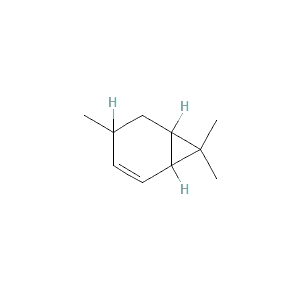 |
| 8 | Hexanoic acid, TMS derivative | 6.82 | 1883.4 | C_9_H_20_O_2_Si | 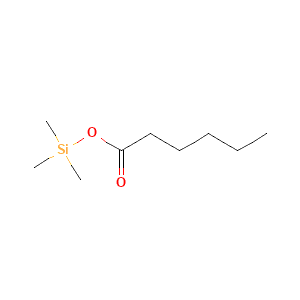 |
| 9 | Benzenamine, N,N-dimethyl- | 6.96 | 121.18 | C_8_H_11_N | 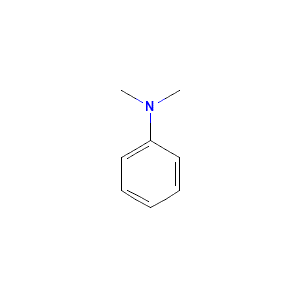 |
| 10 | Tetradecane, 1-chloro- | 7.33 | 232.83 | C_14_H_29_Cl | 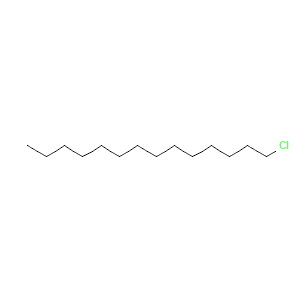 |
| 11 | Cyclomethicone 5 | 7.79 | 370 | C_10_H_30_O_5_Si_5_ | 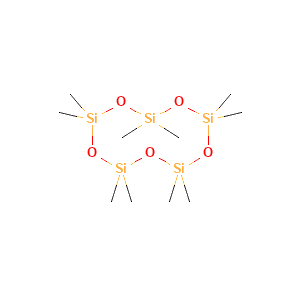 |
| 12 | Eugenol | 13.49 | 164 | C_10_H_12_O_2_ | 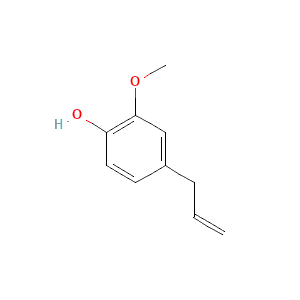 |
| 13 | Nerol | 14.3 | 154 | C_10_H_18_O | 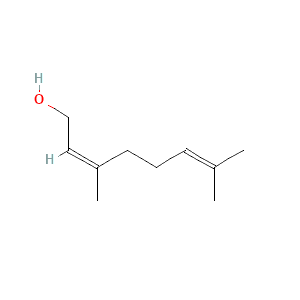 |
| 14 | 4,6,8-Trimethyl-1-nonene | 17.27 | 168 | C_12_H_24_ | 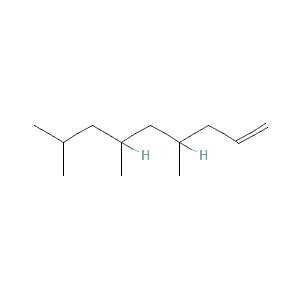 |
| 15 | 2,5-Di-tert-butyl-phenol | 17.67 | 206 | C_14_H_22_O | 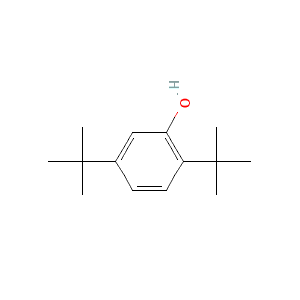 |
| 16 | Diethyl phthalate | 19.38 | 222 | C_12_H_14_O_4_ | 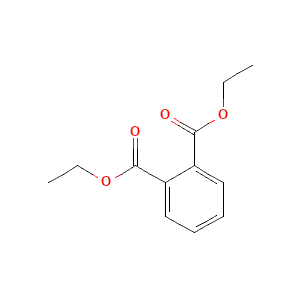 |
| 17 | Tricosane | 19.67 | 324 | C_23_H_48_ | 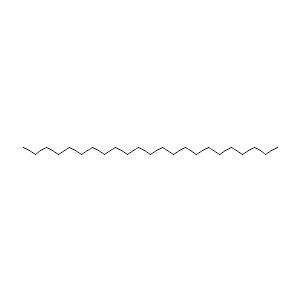 |
| 18 | Gabapentin | 35.10 | 329 | C_16_H_27_NO_6_ | 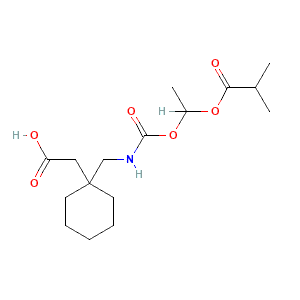 |
| 19 | Quercetin 7,3',4'-trimethyl ether | 37.68 | 344 | C_18_H_16_O_7_ | 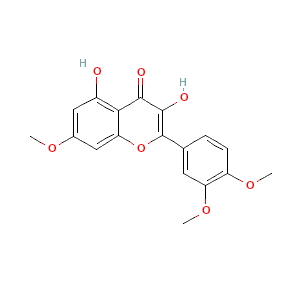 |
| 20 | 2-Monostearin, 2TMS derivative | 40.17 | 509.2 | C_27_H_58_O_4_Si_2_ | 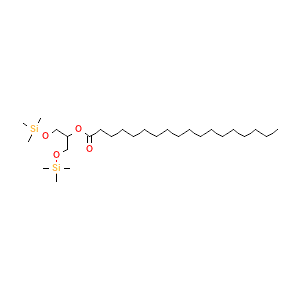 |
| 21 | 9-dexoso-9-x-acetoxy-3,8,12-tri-O-acetytingol | 40.8 | 536.6 | C_28_H_40_O_10_ | 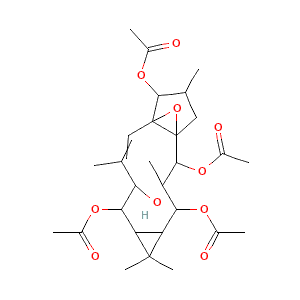 |
| 22 | Phenol, 2,4-bis(1,1-dimethylethyl)-, phosphite (3:1) | 48.33 | 646.9 | C_42_H_63_O_3_P | 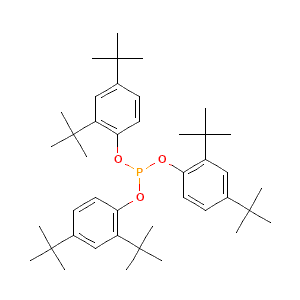 |
| 23 | 2-methylene-4,8,8-trimethyl-4-vinyl-bicyclo[5.2.0]nonane | 14.94 | 204.35 | C_15_H_24_ | 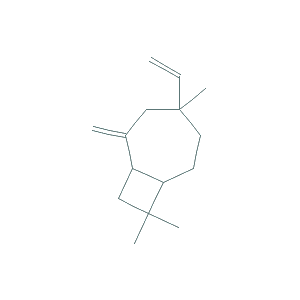 |
| 24 | 5-Isopropenyl-2-methyl-2-cyclohexen-1-yl pivalate | 15.31 | 236.35 | C_15_H_24_O_2_ | 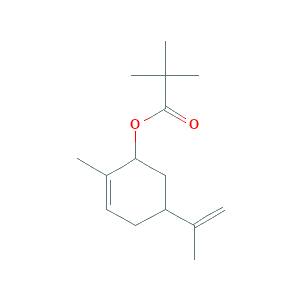 |
| 25 | 1,2-Dibromododecane | 20.44 | 328.13 | C_12_H_24_Br_2_ | 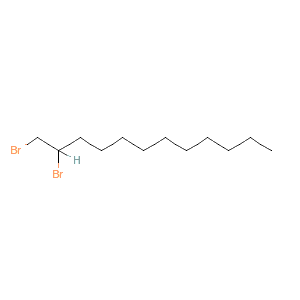 |
| 26 | 2-(Octadecyloxy)ethanol | 22.96 | 314.5 | C_20_H_42_O_2_ | 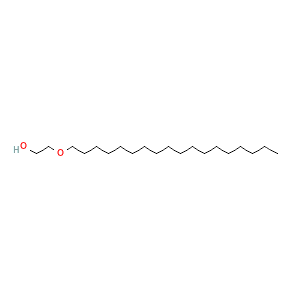 |
| 27 | Tetradecanoic acid | 25.04 | 300.6 | C_17_H_36_O_2_Si | 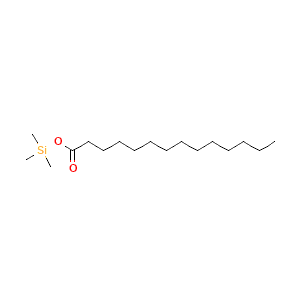 |
| 28 | n-Pentacosane | 26.33 | 352.7 | C_25_H_52_ | 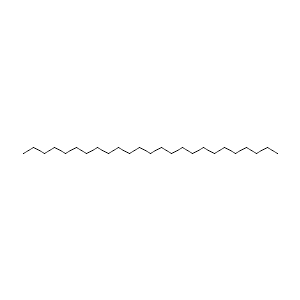 |
| 29 | tert-Hexadecanethiol | 30.80 | 969.5 | C_48_H_99_AuS_3_ | 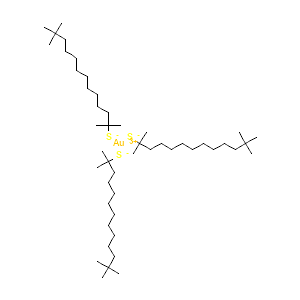 |
| 30 | Monopalmitin | 31.11 | 330.5 | C_19_H_38_O_4_ | 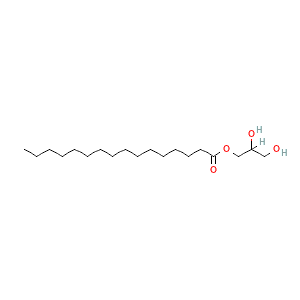 |
| 31 | 2-bromooctadecanal | 34.30 | 347.4 | C_18_H_35_BrO | 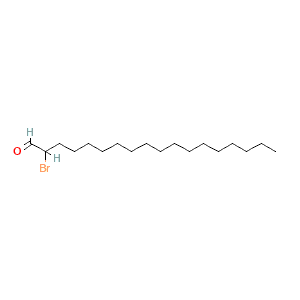 |
| 32 | [(Z)-7-methyltetradec-8-enyl] acetate | 34.61 | 268.4 | C_17_H_32_O_2_ | 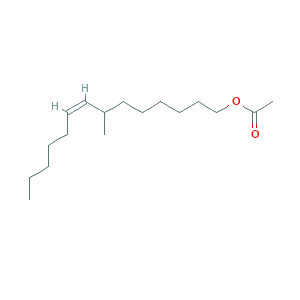 |
