# Supplementary figures and images for: Biochemical and functional characterization of heat-inactivated coelomic fluid from earthworms as a potential alternative for fetal bovine serum in animal cell culture
Source: Sci Rep. 2024 Mar 7;14:5606. doi: 10.1038/s41598-024-56169-0 (PMC10920628; doi:10.1038/s41598-024-56169-0)

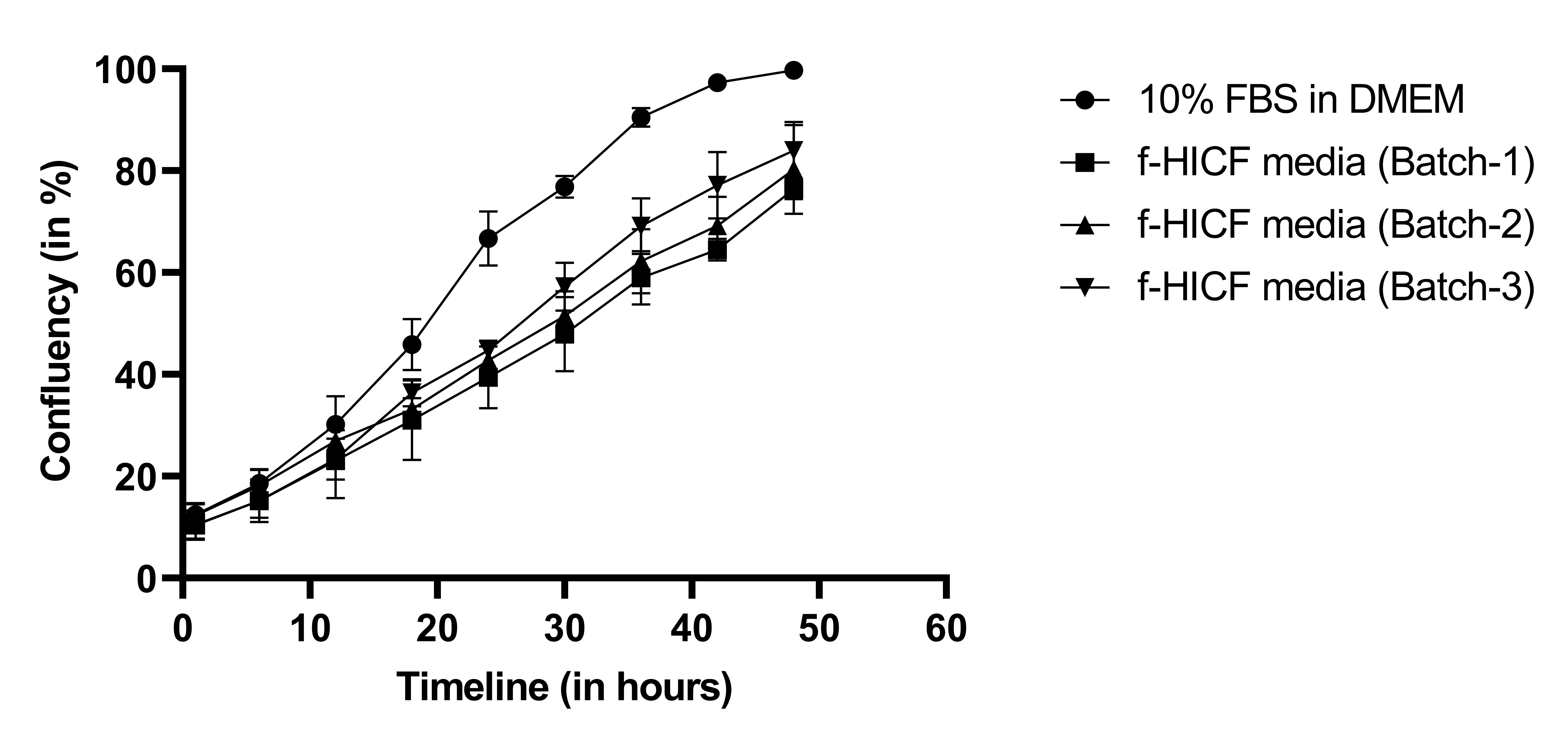

Supplement: Supplementary file 2 — Dataset S2. [file 41598_2024_56169_MOESM2_ESM.tif]
